# Autophagy Induced by Proteasomal DUB Inhibitor NiPT Restricts NiPT-Mediated Cancer Cell Death

**DOI:** 10.3389/fonc.2020.00348

**Published:** 2020-03-27

**Authors:** Jinghong Chen, Xin Chen, Dacai Xu, Li Yang, Zhenjun Yang, Qianqian Yang, Ding Yan, Peiquan Zhang, Du Feng, Jinbao Liu

**Affiliations:** ^1^Translational Medicine Center, The Second Affiliated Hospital of Guangzhou Medical University, Guangzhou, China; ^2^Affiliated Cancer Hospital and Institute of Guangzhou Medical University, Guangzhou Municipal and Guangdong Provincial Key Laboratory of Protein Modification and Degradation, State Key Laboratory of Respiratory Disease, School of Basic Medical Sciences, Guangzhou Medical University, Guangzhou, China; ^3^The Department of Physiology, School of Basic Medical Sciences, Guizhou Medical University, Guizhou, China

**Keywords:** UPS, autophagy, p62, LC3, DUBs, metal-based compounds, anticancer therapy, NiPT

## Abstract

Ubiquitin–proteasome system (UPS) and autophagy–lysosome pathway (ALP) are two major systems for protein quality control (PQC) in eukaryotic cells. Interconnectivity between these two pathways has been suggested, but the molecular detail of how they impact each other remains elusive. Proteasomal deubiquitinase (DUB) is an important constituent in the UPS and has proved to be a novel anticancer target. We have previously found that a novel DUB inhibitor, nickel complex NiPT, induces apoptosis in both cultured tumor cell lines and cancer cells from acute myeloid leukemia human patients. In this study, we found that NiPT triggered autophagy both *in vitro* and *in vivo*. Mechanistically, NiPT targets two DUBs, USP14, and UCHL5, and increased the total cellular level of polyubiquitination. Deletion of the Ubiquitin Associated (UBA) domain of P62 that is required for polyubiquitin binding prevented NiPT-induced autophagy. NiPT-induced autophagy is through either concomitant activation of AMP-activated protein kinase (AMPK) and inhibition of mechanistic target of rapamycin (mTOR) signaling, or eliciting endoplasmic reticulum (ER)-stress by activating activating transcription factor 4 (ATF4) and C/EBP-homologous protein (CHOP). Moreover, NiPT could induce more lung cancer cells undergoing apoptosis if it synergistically uses autophagy inhibitors, suggesting that NiPT-induced autophagy protects cancer cell from death. Collectively, our findings demonstrate that autophagy inhibition enhances the anticancer effects of proteasomal DUB inhibitor and might be an effective treatment strategy for lung cancer.

## Introduction

Ubiquitin–proteasome system (UPS) is the main proteolytic pathway for short-lived, misfolded, and damaged proteins in cells, and are involved in the degradation of more than 80% of proteins in cells (1, 2). UPS-mediated protein degradation is a multistep reaction process with a variety of different proteins involved (3). Specifically, the protein to be degraded is first labeled by ubiquitin and then recognized and degraded by proteasome. Through such a process that requires energy consumption, cells degrade unwanted proteins in a highly specific way (4). In addition to being a garbage disposal biological machinery, UPS regulates a series of important biological processes within cells such as cell proliferation, apoptosis, signal transduction, and immunity, and the deregulation of UPS is closely related to the pathogenesis of diseases (5–7). Studies confirmed that UPS has important pathophysiological significance in cardiovascular disease, which can regulate the occurrence and development of important diseases such as atherosclerosis, reperfusion injury after ischemia, familial cardiomyopathy, myocardial hypertrophy, and heart failure (6).

The autophagy–lysosome pathway (ALP) identifies and destructs large and potentially dangerous cellular components including large protein aggregates and dysfunctional or unwanted organelles and has emerged as a crucial adaptive mechanism to handle various cellular stresses such as nutrient deprivation, hypoxia, and oxidative stress (8–14). Importantly, both UPS and ALP are protein quality control systems and they communicate with each other in different ways (1, 5). Several recent studies have reported the involvement of both AMP-activated protein kinase (AMPK) and mechanistic target of rapamycin 1 (mTORC1) when proteasome activity is impaired. In some cancer cell lines such as human breast, cervical, and lung cancer cells, proteasome inhibitors indirectly activate AMPK primarily via Ca^2+^-CaMKKβ-dependent pathway (15, 16), whereas in macrophages and epithelial and endothelial cells, inhibition of the 26S proteasome alters cellular bioenergetics and redox status, leading to immediate activation of AMPK (17). In addition, proteasome impairment will eventually affect the function of all other organelles. Proteasome inhibitors nelfinavir and bortezomib induce endoplasmic reticulum (ER) stress by inhibiting mTOR activity via activating transcription factor (ATF)-mediated sestrin-2 regulation (18). Li et al. reported a FUNDC1/HSP70-dependent mitochondrial proteostatic stress pathway in which excessive accumulation of unfolded proteins upon proteasome inhibition on the mitochondria impairs mitochondrial integrity and activates AMPK (19). Observations in the above reports all suggested the activation of AMPK and inhibition of mTOR upon loss of function of proteasome. However, Rui et al. provided inconsistent data that under nutrient-rich conditions, inactivation of proteasome function induced an autophagy-dependent process and relied on the critical autophagy components ATG7 and ULK1, but is not involved the upstream autophagy regulators MTORC1 or AMPK. Further, the authors demonstrated that it otherwise depends on ER stress pathway (20).

Deubiquitination is the reverse process of ubiquitination accomplished by deubiquitinases (DUBs) that remove polyubiquitin chains from target proteins (21). Polyubiquitinated substrates are recognized, unfolded, and deubiquitinated by the 19S proteasome. Because of the vital role of DUBs, some of its important component proteins have become drug targets, a large category of which is DUB inhibitor, such as B-ap15, tricyclic heterocyclics, WPI 130, Azepan-4-ones, and PX-478 (22, 23). An iconic achievement in the field of metal-based anticancer drugs was the discovery of the biological activity of cisplatin (24). At present, this type of drug is still widely used in clinical treatment of breast, neck, lung, ovarian, and testicular cancer. However, one of the biggest drawbacks of cisplatin is that its side effects are too large, so finding new, metal-based compounds with less side effects has become the future direction for anticancer drugs. Recently, we and others have discovered a variety of metal-based compounds, such as copper-based proteasome inhibitors (CuPT) (25), gold complexes (auranofin and AUIII) (26), and pyrithione–metal chelates (ZnPT, PtPT, and NiPT) (24). PtPT and NiPT are our newly developed DUB inhibitors, which target 19S proteasomal DUBs, UCHL5 and USP14, without inhibiting 20S proteasome activities (27, 28). However, whether this new type of DUB inhibitor can regulate autophagy has never been studied.

Here, we performed a series of screening of these metal-based compounds and found that most of them are able to induce autophagy, among which PtPT, PdPT, CuPT, and NiPT showed more effectiveness. Because NiPT has less cell death-inducing effect, we chose NiPT as the representative compound in the present study. We found that NiPT triggered autophagy in both A549 and H1299 lung cancer cell lines and in xenograft lung tumor in nude mice. NiPT targets two DUBs, USP14 and UCHL5, and increased the level of polyubiquitination of autophagy receptor P62 and total cellular level of ubiquitins. Deletion of the UBA domain of P62 that is required for its polyubiquitin binding impaired NiPT-induced autophagy. NiPT-induced autophagy is through concomitant activation of AMPK and inhibition of mTOR signaling, as well as eliciting ER-stress by activating ATF4 and C/EBP-homologous protein (CHOP). Moreover, NiPT increased the apoptosis of lung cancer cells with synergistical use of autophagy inhibitors, suggesting that NiPT-induced autophagy protects cancer cell from death. Thus, our results suggest combinational administration of DUBs inhibitors with autophagy–lysosome inhibitors may benefit anticancer treatment.

## Results

### The Metal Ion Complex NiPT Induces Autophagy in A549 and NCI-H1299 Lung Cancer Cells and in Solid Tumor of Nude Mice

**Figure 1:**
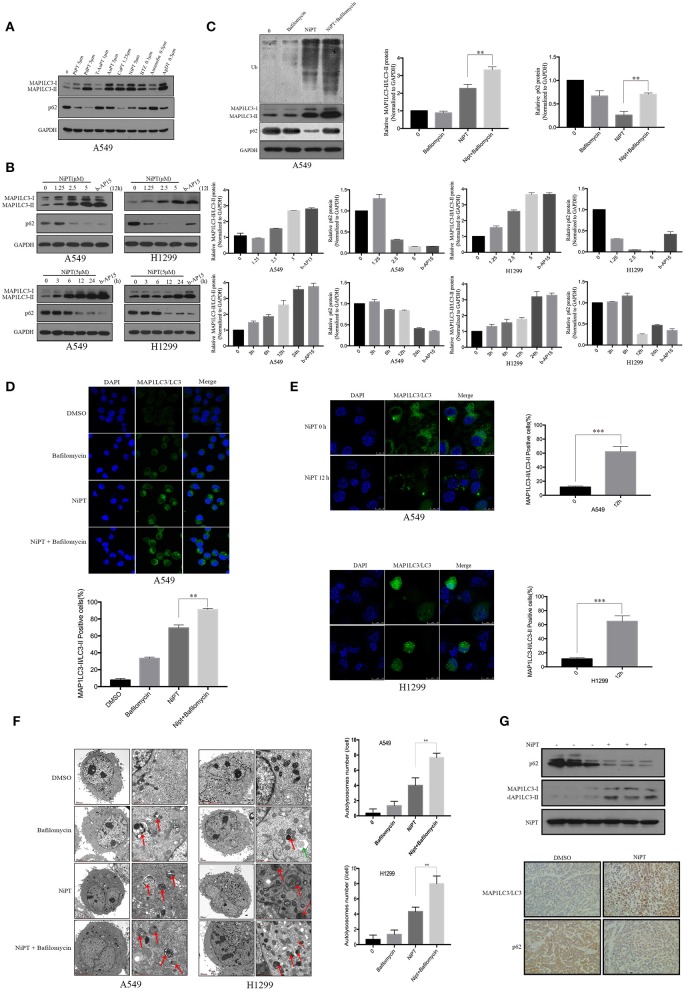
The metal ion complex NiPT induces autophagy in A549 and NCI-H1299 lung cancer cells and in solid tumor of nude mice. **(A)** A549 cells were treated with or without various metal ion complexes for 12h. The autophagy marker proteins LC3 and P62 were analyzed by immunoblotting. **(B)** A549 and NCI-H1299 cells were treated with various concentrations of NiPT for 12 h or with 5 μM NiPT for the indicated time points and 1 μM b-AP15 (proteasome inhibitor, positive control) for 12 h. The protein levels of LC3 and P62 were analyzed by immunoblotting. Bar graphs represent the relative MAP1LC3/LC3-II and SQSTM1/p62 protein levels normalized to that of Glyceraldehyde 3-phosphate Dehydrogenase (GAPDH) of different groups (*P < 0.05, **P < 0.001, *t*-test, *n* = 3). **(C)** A549 cells were treated with or without 5 μM NiPT in the presence or absence of bafilomycin (100 nM) for 12 h. The expression levels of ubiquitin, LC3, and P62 were analyzed by immunoblotting. Bar graphs represent the relative MAP1LC3/LC3-II and SQSTM1/p62 protein levels normalized to that of GAPDH of different groups (*P < 0.05, **P < 0.001, *t*-test, *n* = 3). **(D)** A549 cells were treated with or without 5 μM NiPT in the presence or absence of 100 nM bafilomycin for 12 h. Endogenous green LC3 was analyzed by confocal microscopy (630×). Bar graphs represent the percentage of Endogenous LC3-positive cells in control or NiPT-treated group (*P < 0.05, **P < 0.001, *t*-test, *n* = 3, bars represent SEM, cells containing more than 5 foci were scored as positive and 30 cells were analyzed per experiment). **(E)** A549 and NCI-H1299 cells were transiently transfected with YFP-LC3 plasmids. Cells were treated with or without 5 μM NiPT for 12 h. YFP-LC3 dots were analyzed by confocal microscopy (630×). Bar graphs represent the percentage of YFP- LC3-positive cells in control or NiPT -treated group (*P < 0.05, **P < 0.001, *t*-test, *n* = 3, bars represent SEM. Cells containing more than 5 foci were scored as positive, and 30 cells were analyzed per experiment). **(F)** A549 and NCI-H1299 cells were treated with 5 μM NiPT, 100 nM bafilomycin for 12 h. Cells were subjected to electron microscopy analysis. The green arrow indicates autophagosomes (AP) and the red arrows indicate autolysosomes (AL). Left scale bar, 2 μm; scale bar in magnified pictures, 0.5 μm. The number of autophagosome-like structures in each cell was quantitated (**P < 0.001, *t*-test, *n* = 3, bars represent SEM). **(G)** The expressions of LC3 and P62 were detected by immunoblotting in tumor tissue (left panel). Representative immunohistochemical staining for LC3 and P62 in A549 xenograft tumors in mice treated with vehicle or NiPT (100×) (right panel). Tumor volumes were calculated by the following formula: a2 × b × 0.4, where a is the smallest diameter and b is the diameter perpendicular to a. The animals were then euthanized, and tumor xenografts were immediately removed, weighed, and frozen or fixed for biochemical or histological analyses, respectively.

LC3 and P62 (also known as SQSTM1) are two established markers of autophagy (29, 30). To evaluate the effects of the metal ion complex on autophagy, we first screened a group of metal ion complexes such as PtPT, PdPT, T-AuPT, AuPT, CuPT, NiPT, BTZ, auranofin, and AgDT in A549 cells. All metal ion complexes exerted autophagy-inducing activity as evidenced by the increased LC3 I to LC3 II transition and the decreased autophagy substrate p62 level, except for T-AuPT, AuPT, and auranofin, which demonstrated weak autophagy-inducing effects (Figure 1A). As PdPT, CuPT, and NiPT showed almost the same autophagy-induction activity and NiPT showed the lowest cytotoxicity, we chose NiPT as the representative metal ion complex to further study its autophagy-inducing mechanism. As shown in Figure 1B, NiPT promoted LC3 I to LC3 II transition and p62 degradation in both A549 and H1299 lung cancer cell lines in a dose- and time-dependent manner. LC3 and P62 degradation induced by NiPT can be reversed by lysosome inhibitor bafilomycin A1 (Baf) to some extent (Figure 1C), indicating that NiPT indeed promotes autophagy.

Immunofluorescence demonstrated that NiPT could potently induce LC3 puncta formation, as compared to control (Figures 1D,E). Electron microscopy further verified that NiPT induced the formation of autolysosome-like structures in both cell types (Figure 1F).

We then evaluated the role of NiPT in autophagy *in vivo* and found that NiPT could significantly promote autophagy in solid tumor of nude mice, as evidenced by increased degradation of p62 and the elevated expression of LC3-I and II (Figure 1G, upper panel). Similarly, immunohistochemistry demonstrated that p62 level was remarkably reduced and LC3 II staining was significantly enhanced by NiPT in the xenograft solid tumor in nude mice (Figure 1G, lower panel).

### NiPT Inhibits DUBs USP14 and UCHL5, and Promotes the Cytosolic Ubiquitin Level

**Figure 2:**
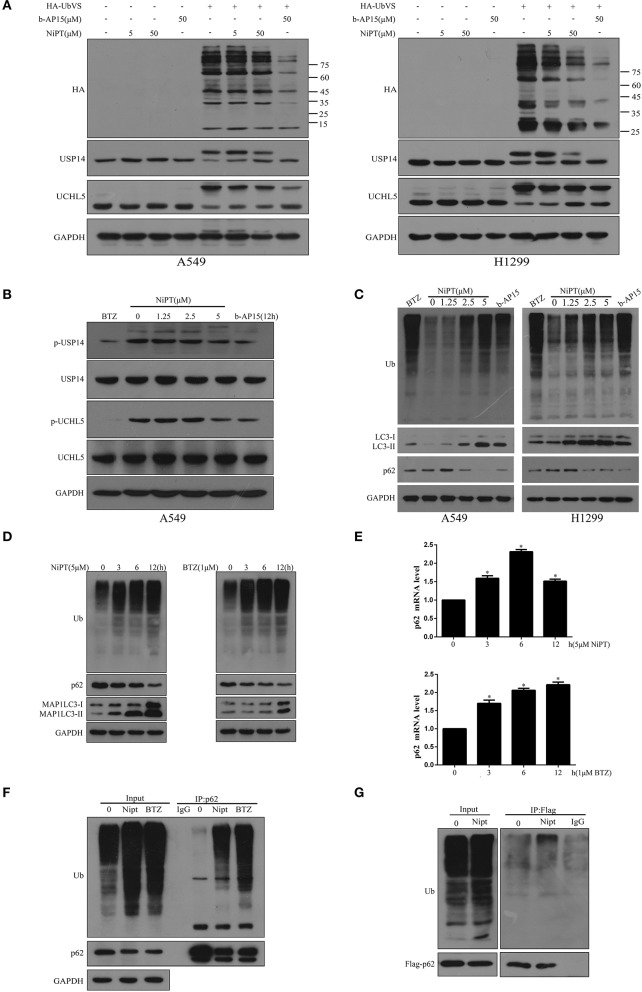
NiPT inhibits DUBs USP14 and UCHL5, and promotes the cytosolic ubiquitin level. **(A)** Active-site–directed labeling of proteasomal DUBs. A549 and NCI-H1299 cells were treated with NiPT (0, 5, 50 μM), and 50 μM b-AP15 followed by labeling with or without HA-UbVS, and then examined by immunoblotting. **(B)** A549 cells were treated with various concentrations of NiPT, bortezomib (BTZ, 100 nM), and b-AP15(1 μM) for 12 h. The expression levels of p-USP14, USP14, p-UCHL5, and UCHL5 were analyzed by immunoblotting. **(C)** A549 and NCI-H1299 cells were treated with various concentrations of NiPT,100 nM bortezomib, and 1 μM b-AP15 for 12 h. The expression levels of LC3, P62, and Ub were analyzed by immunoblotting. **(D)** A549 cells were treated with 5 μM NiPT or 1 μM bortezomib for the indicated periods. The expression levels of Ub, LC3, and P62 were analyzed by immunoblotting. **(E)** A549 cells were treated with 5 μM NiPT or 1 μM bortezomib for the indicated periods. The expression levels of P62 mRNA were analyzed by Real-time Quantitative Polymerase Chain Reaction (RT-PCR). **(F)** Endogenous P62 is ubiquitinated. Ubiquitinated proteins were immunoprecipitated with antibody to ubiquitinated P62 from A549 cells after 5 μM NiPT or 1 μM bortezomib for 9 h treatment as indicated. **(G)** Exogenous P62 is ubiquitinated. Flag-tagged P62 was immunoprecipitated with antiflag antibody from A549 cells treated with or without 5 μM NiPT for 9 h.

Then, we asked if NiPT could target DUBs. 0, 5, or 50 μM NiPT was subjected to A549 and H1299 cells with or without HA-UbVS, respectively. As shown in Figure 2A, HA-UbVS strongly binds to both USP14 and UCHL5 in untreated cells, whereas the binding of HA-UbVS to USP14 and UCHL5 is weakened in the presence of NiPT in both A549 and H1299 cells, but to a less extent to the b-AP15-treated positive control cells. Previous reports showed that USP14 and UCHL5 are constitutively phosphorylated under normal conditions (31, 32). Here, we observed that 5 μM NiPT caused the dephosphorylation of USP14 and UCHL5 at 12 h, which is similar to BTZ or b-AP15, two established proteasome deubiquitinase inhibitors (33, 34) (Figure 2B). Because NiPT caused P62 degradation, we tested whether NiPT-induced P62 degradation is followed by ubiquitin accumulation. Consistent with BTZ or B-AP15, NiPT increased the cellular accumulation of ubiquitin in a dose-dependent manner, reaching to the highest effects at 5 μM, whereas the P62 level firstly increased at 1.25 μM, and then decreased at 2.5 and 5 μM (Figure 2C). As 5 μM NiPT has the largest effect to induce autophagy, we then tested it in a time course. We found that NiPT-induced ubiquitin accumulation is time-dependent and closely associated with autophagy activity (Figure 2D). The reduced P62 level is not because of the suppression of the P62 transcription since the P62 mRNA level is higher than that in control cells upon NiPT treatment (Figure 2E). In addition, immune-precipitation assay verified that both endogenous P62 and overexpressed Flag-P62 are able to pull down a large amount of ubiquitin complexes in cells administrated by NiPT (Figures 2F,G).

### P62 Interacts With USP14 and UCHL5 and Deletion of the UBA Domain of P62 Promotes Autophagy Induced by NiPT

**Figure 3:**
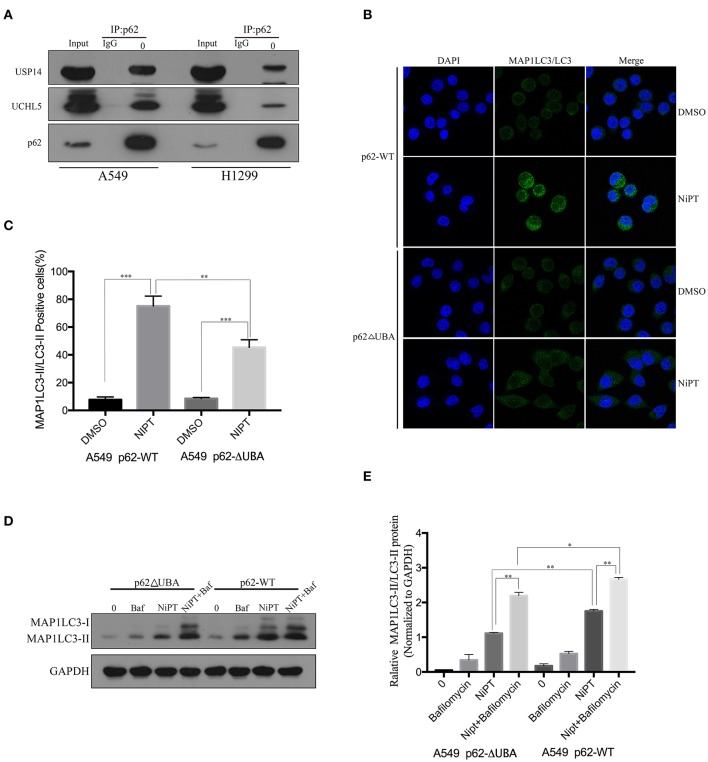
P62 interacts with USP14 and UCHL5, and deletion of the UBA domain of P62 promotes autophagy induced by NiPT. **(A)** Endogenous P62 was immunoprecipitated from A549 or NCI-H1299 cells, and USP14, UCHL5, and P62 were blotted by indicated antibodies, respectively. **(B)** A549 cells were transiently transfected with Flag-P62-ΔUBA or Flag-P62-WT plasmids. Cells were treated with or without 5 μM NiPT for 12 h. Endogenous LC3 (green) was stained and analyzed by confocal microscopy (630×). Bar graphs represent the percentage of endogenous LC3-positive cells in control or NiPT-treated group in **(C)** (*P < 0.05, **P < 0.001, *t*-test, *n* = 3, bars represent SEM. Cells containing more than 5 foci were scored as positive, and 30 cells were analyzed per experiment). **(D)** A549 cells were transiently transfected with Flag-P62-ΔUBA or Flag-P62-WT plasmids. Cells were treated with or without 5 μM NiPT in the presence of absence of 100 nM bafilomycin for 12 h. The expression level of LC3 was analyzed by immunoblotting. **(E)** Bar graphs represent the relative MAP1LC3/LC3-II and SQSTM1/p62 protein levels in **(D)** normalized to that of GAPDH of different groups (*P < 0.05, **P < 0.001, *t*-test, *n* = 3).

Because NiPT promotes the degradation of p62, which is closely associated with the accumulation of ubiquitin, we asked if p62 has a direct relationship with USP14 and UCHL5. Unlike IgG control, anti-p62 antibody immune-precipitated both USP14 and UCHL5 in A549 and H1299 cells (Figure 3A). As p62 has a ubiquitin-binding domain (UBA), which may contribute to the ubiquitin accumulation upon inhibition of the DUBs, we examined if the deletion of the UBA domain will affect the autophagy induced by NiPT. Indeed, NiPT successfully promoted LC3 puncta formation in cells bearing full-length p62, whereas it failed to induce autophagy in cells with overexpressed p62 (delta UBA) (Figures 3B,C). Similarly, the LC3 I to LC3 II transition and the P62 degradation were observed in NiPT-treated cells expressing full-length P62. However, cells with over-expressed p62 (delta UBA) had less transited LC3-II (Figures 3D,E).

### NiPT Induces Autophagy Through AMPK-mTOR and ER Stress Pathways in Lung Cancer Cells

**Figure 4:**
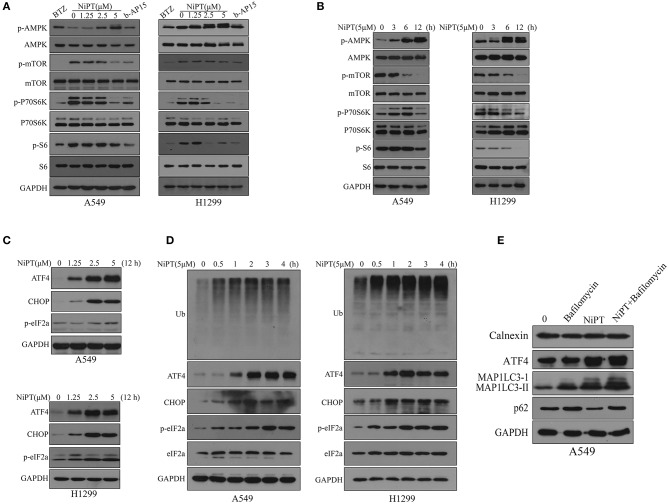
NiPT induces autophagy through AMP-activated protein kinase–mechanistic target of rapamycin (AMPK-mTOR) and endoplasmic reticulum (ER) stress pathways in lung cancer cells. **(A)** A549 and NCI-H1299 cells were treated with various concentrations of NiPT, 100 nM bortezomib, and 1 μM b-AP15 for 12 h. The expression levels of p-AMPK, p-mTOR, p-P70S6K S6, and p-S6 were analyzed by immunoblotting. **(B)** A549 and NCI-H1299 cells were treated with 5 μM NiPT for the indicated time points. The expression levels of p-AMPK, p-mTOR, p-P70S6K S6, and p-S6 were analyzed by immunoblotting. **(C)** A549 and NCI-H1299 cells were treated with various concentrations of NiPT for 12 h. The expression levels of ATF4, C/EBP-homologous protein (CHOP), and p-eIF2a were analyzed by immunoblotting. **(D)** A549 and NCI-H1299 cells were treated with 5 μM NiPT for the indicated time points. The expression levels of ATF4, CHOP, and p-eIF2a were analyzed by immunoblotting. **(E)** A549 cells were treated with or without 5 μM NiPT and bafilomycin (10, 50, 100 nM) for 12 h. The expression levels of calnexin, ATF4, LC3, and p62 were analyzed by immunoblotting.

Next, we investigated the molecular mechanism by which NiPT induces autophagy. Upon induction of autophagy, AMPK is phosphorylated and activated, which, in turn, inhibits mTOR and lifts the repression of ULK1, the key kinase for autophagy initiation (35, 36). NiPT effectively promoted the phosphorylation of AMPK and otherwise dephosphorylation of mTOR, therefore suppressing P70S6K, the substrate of mTOR, in both cell lines in a dose-dependent manner (Figure 4A). As 5 μM NiPT reached the maximal inhibition of mTOR and activation of AMPK, we fixed the concentration at 5 μM and evaluated the effect in a prolonged time of incubation. NiPT induced a dramatic activation of AMPK and abolished the phosphorylation of mTOR and its downstream signaling at 12 h in both cell types (Figure 4B).

Free intracellular ubiquitins will be exhausted when proteasome is inhibited, which may therefore lead to ER stress (37, 38). Autophagy is thought to be activated and involved in ER stress to fast degrade a vast amount of unfolded proteins produced in ER stress (1, 38). We thus investigated if NiPT activated ER stress pathway. NiPT induced a significant ER stress response in both A549 and H1299 cell lines as evidenced by the increased expression of ER stress markers, ATF4 and CHOP, and phosphorylation of eIF2a (39, 40) (Figures 4C–D). As 5 μM NiPT has the strongest effect to induce ER stress response, we fixed the concentration of NiPT at 5 μM and evaluated its effects at different time points (Figure 4D). NiPT promoted ER stress in a time-dependent manner with increased level of cellular ubiquitins (Figure 4D). Of note, we did not observe ER-phagy in these cells treated with NiPT, as ER marker Calnexin did not change at all (Figure 4E).

### Inhibition of Autophagy Will Aggravate Apoptosis of Lung Cancer Cells Induced by NiPT

**Figure 5:**
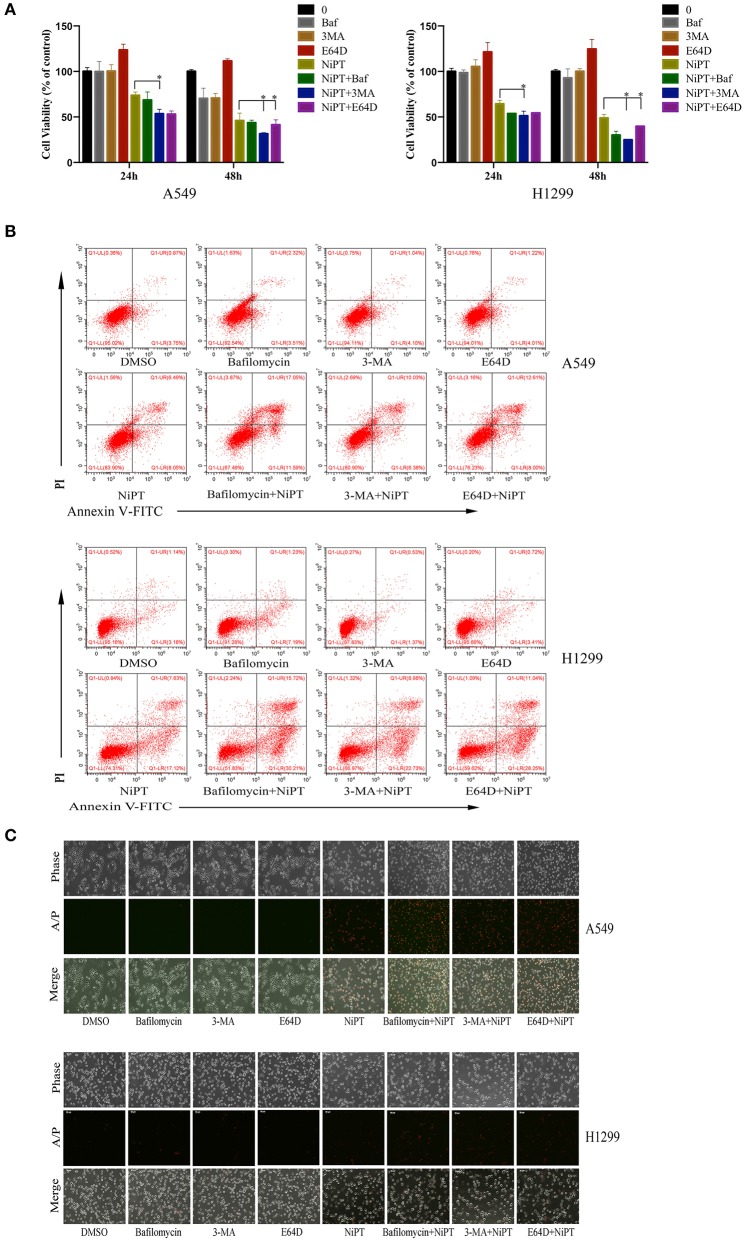
Inhibition of autophagy will aggravate cell death of lung cancer cells induced by NiPT A549 and NCI-H1299 cells were treated with or without 5 μM NiPT in the presence of 100 nM bafilomycin, 3MA, or E64D, respectively. **(A)** Cell viability was detected by 3-(4,5-dimethylthiazol-2-yl)-2,5-diphenyltetrazolium bromide (MTS) assay. Data from three repeats. Mean+SD (*n* = 3). **(B)** Cells were stained with AnnexinV/PI and examined with flow cytometry. **(C)** Cells were stained with AnnexinV/PI and imaged under a fluorescent microscopy.

Accumulative evidences have shown that deubiquitinase inhibitors suppress the growth of cancer cells *in vitro* and *in vivo* (6, 25, 28, 31). Since the data above indicated that NiPT could induce autophagy, we next examined if suppressing autophagy will benefit the NiPT-induced cell death. In the presence of NiPT, the cell viability of both A549 and H1299 cells reduced after 24 or 48 h if autophagy was synergistically suppressed by autophagy inhibitors 3-MA, Bafinomycin, or E64D in these cells (Figure 5A). These observations were further corroborated by cell apoptosis assays. As shown in Figure 5B, the number of apoptotic cells (including those cells in both early and late stages of apoptosis) increased from 4.62% (control) to 14.54% (with NiPT) in A549 cells, or from 8.77% (control) to 24.75% (with NiPT) in H1299 cells, respectively. More importantly, the number of apoptotic cells was more considerably increased in both cell types if these cells were synergistically treated with autophagy inhibitors. Consistently, the cell death rate is higher in cells subjected with both NiPT and autophagy inhibitors than that in NiPT alone by phase contrast microscopy (Figure 5C). The responsiveness of autophagy to NiPT occurred much earlier than apoptosis in both A549 and H1299 cells, as indicated by LC3-II (1 h) and Poly (ADP-ribose) polymerase (PARP) cleavage (8 h) (Supplementary Figures 1A–D). However, the PARP cleavage occurred earlier to 3 h, if autophagy essential gene *atg7* has been deleted in these cells (Supplementary Figures 1E,F), indicating that the cellular protective mechanism autophagy is also triggered by NiPT when it kills cancer cells in the meantime.

### NiPT Inhibits Tumor Growth *in vivo*

**Figure 6:**
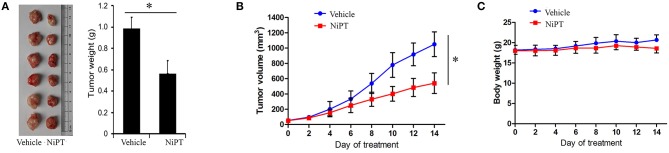
NiPT inhibits tumor growth *in vivo*. **(A–C)** Bagg Albino/C (BALB/c) nude mice bearing A549 tumors were treated with vehicle or NiPT (40 mg/kg/day, p.o.) for 14 days. Tumor size was observed every other day. Body weight, tumor volume, and tumor images were shown.

To examine the functional role of NiPT *in vivo*, we administrated NiPT to nude mice with established solid tumor and monitored the tumor size from 0 to 14 days. Tumor volume began to dramatically reduce after 6 days of treatment in contrast to vehicle control-treated mice (Figure 6A). In addition to reducing the tumor size, NiPT also promoted the reduction of the tumor weight in these mice (Figure 6B), whereas the body weight does not change too much in these mice (Figure 6C). Taken together, our data demonstrated that NiPT could effectively inhibit tumor growth *in vivo*.

### Discussion

We have been working on high-efficiency and low-toxicity metal complexes for many years. We used PT as the ligand, to synthetize a variety of metal complexes, such as CuPT, PtPT, and NiPT (24, 25, 31). Our previous studies have shown that they have good antitumor efficacy *in vitro* and *in vivo* via inhibiting the deubiquitinase activity of 26S proteasome. Based on this, we synthesized a number of metal complexes and applied them to lung cancer cells. We found that the metal complexes PtPT, AuPT, CuPT, NiPT, and AgDT can induce autophagy in lung cancer cells. In this study, the nickel–ion complex NiPT was selected to explore the mechanism of action on autophagy according to its efficacy and toxicity.

P62 is an interesting protein because it has both UBA domain, which binds to ubiquitinated protein substrate, and LIR (LC3 interaction region), which interacts with LC3 (41–43). This makes P62 an interesting and important protein that mediates the crosstalk between the UPS system and autophagy. For example, P62 mediates the autophagic degradation of the E3 ligase Keap1 or directly controls the proteasome abundance by proteaphagy (44, 45). Nontheless, proteasomal degradation of ULK1 and other autophagy regulators could also terminate autophagy (46). Interestingly, the notable common feature of both degradation systems is the use of ubiquitination as a labeling system for their substrates. Ub depletion can be caused by translational inhibitors as well as overwhelmingly non-degradable highly ubiquitinated protein aggregates, which have often sequestered by P62 (1). Polyubiquitin genes and DUB activity are responsible for supplying free Ub and are essential for cellular function and stress tolerance. In line with this, our study found that NiPT can cause the accumulation of cellular ubiquitin by inhibiting the enzyme activity of the two Dubs, UCHL5 and USP14. Likely, bortezomib also causes the accumulation of ubiquitins in lung cancer cell A549, but P62 does not appear to increase with bortezomib in HeLa cells as reported by others (47), but decrease with bortezomib, indicating that there are variable mechanisms underneath the different DUB inhibitors. We also found that P62 protein level decreased gradually in a dose- and time-dependent manner in NiPT-induced lung cancer cells, but P62 was gradually increased at mRNA levels, indicating that P62 was degraded during autophagy. So what effect does the ubiquitination caused by NiPT have on autophagy? We synthesized the wild-type P62 and the P62-UBA (Delta UBA) plasmids and transfected them into A549 cells, respectively. We found that P62-UBA inhibited LC3 I to LC3 II transition and the degradation of P62 under NiPT-treated cells. Consistently, the number of LC3 puncta was also significantly reduced in P62-UBA plasmid transfected cells, indicating that the UBA region is important for NiPT-induced autophagy.

Accumulating evidence has shown that inhibition of DUBs will lead to UPR (unfolded protein response) and the latter will cause ER stress (38, 48–51). ER stress was reported to be associated with autophagy (52). In the present study, UCHL5 and USP14 were inhibited by NiPT, causing the aggregation of ubiquitinated P62 and the increase of global ubiquitinated proteins, which is consistent with b-AP15 and bortezomib. Likewise, ER stress-related proteins ATF4, CHOP, and p-eIF2a were also found to be gradually increased upon NiPT treatment. AMPK was reported to be activated during ER stress (53–55). It is an energy sensor within the cell, sensing the availability of cellular energy status (56). We found that NiPT causes the ER stress response of lung cancer cells, leading to instability in the amount of intracellular energy, resulting in the activation of AMPK as well as the dephosphorylation of the mTOR, the autophagy suppressor. These events therefore led to the dephosphorylation of the downstream molecule P70S6K and S6K, which eventually induced autophagy. In this study, the cancer cell inhibitory effects were found to be amplified if NiPT application is combined with autophagy inhibitors in the meantime. These data suggested that, apart from the cell death, NiPT also induced the tumor cells to develop an autophagy program to antagonize the anticancer effects by NiPT, which added a more complex layer of regulation of this compound in anticancer therapy.

Taken together, our study provided the theoretical basis for the use of NiPT metal complexes in tumor therapy. It is the first time to demonstrate that NiPT can promote total level of ubiquitination by inhibiting the enzyme activity of the two DUBs, UCHL5 and USP14, and therefore regulate autophagy. Moreover, we found that NiPT-induced autophagy is associated with ER stress–AMPK–mTOR–S6K pathway by which NiPT regulates both autophagy and cell death to affect the progression of tumor.

## Materials and Methods

### Cell Culture

The human lung adenocarcinoma (A549 and NCI-H1299) cell lines were ordered from American Type Culture Collection, and have been stored in liquid nitrogen at our lab. Both cell lines were grown in RMPI1640 medium, supplemented with 10% fetal bovine serum (heat inactivated at 56°C for 30 min), penicillin (100 μg/ml), and streptomycin (100 μg/ml) in a humidified 5% CO2 atmosphere at 37°C.

### Antibodies and Reagents

NiPT, PtPT, PdPT, T-AuPT, Au(PPh_3_)PT, CuPT, and AgDT were synthesized in our laboratory and stored as a 10 mM stock solution in dimethyl sulfoxide (DMSO) at −20°C. Auranofin was purchased from Enzo Life Sciences International, Inc. (Plymouth Meeting, PA) and dissolved in DMSO as a 10 mM, stored at −20°C. Bortezomib (Cell Signaling Technology, Beverly, MA, USA) HA–ubiquitin–vinyl sulfone (HA-Ub-VS), and Ub-AMC (BostonBiochem, Cambridge, MA, USA). Antibodies were purchased from following sources: anti-AMPK, anti- phospho-AMPK, anti-mTOR, anti-phospho-mTOR, anti-P70S6K, anti-phospho-P70S6K, anti-S6, anti-phospho-S6, anti-ATF4, anti-CHOP, anti-phospho-eIF2a, anti-LC3, anti-USP14 (D8Q6S), and anti-PARP (Cell Signaling Technology, Beverly, MA, USA); anti-ubiquitin (P4D1) and anti-SQSTM1/P62(Santa Cruz Biotechnology, Santa Cruz, CA); anti-GAPDH and anti-HA-tag (Bioworld Technology, Nanjing, China); and anti-UCHL5 (Abcam, Cambridge, UK). Anti-phospho-USP14 and anti-phospho-UCHL5 were prepared by the Abgent, Inc. Bafilomycin, 3-MA, and E64D were purchased from Enzo Life Sciences (MA, USA). Lipofectamine 2000 (11668–027), goat anti-rabbit Alexa 488 (997773), and opti-MEM medium (22600050) were purchased from the Invitrogen^TM^.

### Cell Death Assay

Apoptotic rates were carried out by flow cytometry using Annexin V-fluoroisothiocyanate (FITC)/propidium iodide (PI) (Keygen, Nanjing, China) double staining (1). Cells were seeded into 6-well culture plates, treated as NiPT, Bafilomycin, 3MA, and E64D for 24 h, then collected and washed with binding buffer, and then incubated in the working solution for 15 min in dark; cells were washed and resuspended with the binding buffer. Lastly, PI was added immediately and flow cytometric analysis. In addition, cells were submitted to Annexin V/PI staining *in situ*, and then imaged with an inverted fluorescence microscope equipped with a digital camera (Axio Obsever Z1, Zeiss, Germany).

### Immunofluorescence

A549 and NCI-H1299 cells were seeded in 24-well plates containing NUNC ThermanoxTM coverslips. The immunofluorescence assay was performed as previously described. After treatment, the cells were washed briefly with Phosphate-buffered saline (PBS), fixed with a 4% paraformaldehyde fixative solution at room temperature for 20 min, and washed three times in PBS for 5 min each. Then, the cells were permeabilized with 0.25% Triton X-100 (Sigma-Aldrich) for 10 min at 4°C and blocked with 4% BSA (MP Biomedicals) solution for 30 min at 37°C. The cells were incubated at 4°C overnight with the primary antibody (1:300). Then, the cells were washed thrice for 5 min in Phosphate-buffered saline with Triton X-100 (PBST) and incubated with the fluorescent secondary antibody for 1h at room temperature in the dark. 4′,6-diamidino-2-phenylindole (DAPI) was added for 5 min to visualize nuclei in the dark. Then, the cells were washed with PBS and dried. After using an antifluorescence quenching reagent, the coverslips were stored at 4°C. The cells were observed at ×100 magnification using a laser scanning confocal microscope (Olympus, FV-1000). The number of cells analyzed per experiment was 30 cells.

### Plasmids and Transfection

The vector pcDNA3.1, YFP-LC3, and Flag-SQSTM1ΔUBA plasmids were all kindly gifted by Professor Xiaofeng Zhu (State Key Laboratory of Oncology in South China; Cancer Center, Sun Yat-sen University, Guangzhou, China). All plasmid transient transfections were performed using Lipofectamine 2000 according to the manufacturer's instructions.

### Active DUB Labeling Assays

These assays were performed as previously reported (4, 5). Purified 26S proteasomes (25 nM) were incubated with NiPT (5, 50 μM) in DUB buffer for 30 min. Cell lysates (5 μg) from NiPT-treated cancer cells were incubated with HA-UbVs at 37°C for another 1 h. These samples were boiled in the reducing sample buffer and subjected with Western blot analysis, HA-UbVS-labeled DUBs were immunodetected using anti-HA antibodies.

### Tumor Xenograft Analysis

All animal experiments were followed in accordance with the protocols and approved by the Institutional Animal Care and Use Committee of Guangzhou Medical University. Nude Balb/c mice (male, 5-week-old, and 20–23 g) were subcutaneously inoculated with approximately 3 × 10^6^ A549 cells in a total volume of 100 μl in the left armpit. Seventy-two hours after inoculation, the mice were randomly divided into two groups (*n* = 6 for each group), and treated with either vehicle (DMSO, polyethylene glycol 400, and 0.9% NaCl at 1:3:6 volume ratio, oral) or NiPT (40 mg/kg/d, oral) for a total of 14 days. Tumor volumes were determined every 3 days. Tumor volumes were calculated by the following formula: a2 × b × 0.4, where a is the smallest diameter and b is the diameter perpendicular to a. The animals were then euthanized, and tumor xenografts were immediately removed, weighed, and frozen or fixed for biochemical or histological analyses, respectively.

### Immunohistochemistry

Xenograft tumors were fixed in 4% paraformaldehyde (PFA), embedded in paraffin, sectioned, and stained with haematoxylin and eosin. Immunohistochemical staining of paraffin-embedded tumor tissues was performed using P62 (Santa Cruz, 1:100 dilution) and LC3 (Cell Signaling Technology, 1:100 dilution) primary antibodies and the ABC Elite immunoperoxidase kit according to the manufacturers' instructions.

### Immunoblotting and Immunoprecipitation

Whole cell lysates were prepared in RIPA buffer (1×PBS, 1% NP-40, 0.5% sodium deoxycholate, 0.1% SDS) supplemented with 10 mM β-glycerophosphate, 10 mM NaF, 1 mM sodium orthovanadate, 1× Roche Complete Mini Protease Inhibitor Cocktail (Roche, Indianapolis, IN), and 1 mM phenylmethylsulfonyl fluoride (PMSF). Lysates were centrifuged at 13,400 g for 15 min at 4°C. Then, the supernatant fraction was collected. Protein concentrations were determined using the Pierce BCA protein assay kit (Thermo, 1862634). Equivalent aliquots of protein samples (20 to 40 mg) were loaded and electrophoresed on SDS-PAGE gels and then transferred to polyvinylidene fluoride/PVDF membranes. SDS-PAGE, transferring, and immunodetection were performed as previously described (2). To analyze protein interactions, anti-FLAG antibody was incubated with Dynabeads™ Antibody Coupling Kit (Life Technologies, Invitrogen, CA) according to the manufacturer's instructions, and cell lysates were incubated with beads for 3 h at room temperature. Precipitated proteins were washed with PBS-T for three times, eluted by Blue Loading Buffer Pack (Cell signaling technology, USA), loaded on SDS-PAGE, and immunoblotted.

### Statistical Analysis

All assays were performed in triplicate. Data are expressed as the mean ± standard deviation (SD). Two-tailed unpaired Student's *t*-test was used to evaluate the statistical significance of the difference between two groups in all experiments. When the difference among ≥3 groups were evaluated, one-way analysis of variance (one-way ANOVA) was performed. The *p* < 0.05 was considered statistically significant.

## Data Availability Statement

All datasets generated for this study are included in the article/Supplementary Material.

## Ethics Statement

The animal study was reviewed and approved by the affiliation of the ethics committee of Guangzhou Medical University.

## Author Contributions

JL, JC, and DF designed the experiments. JC, XC, and DX performed most of the experiments with the help of LY, ZY, QY, DY, and PZ. JL, JC, and DF wrote the paper.

### Conflict of Interest

The authors declare that the research was conducted in the absence of any commercial or financial relationships that could be construed as a potential conflict of interest.
