# Supplementary material for: Autophagy Induced by Proteasomal DUB Inhibitor NiPT Restricts NiPT-Mediated Cancer Cell Death
Source: Front Oncol. 2020 Mar 27;10:348. doi: 10.3389/fonc.2020.00348 (PMC7119081; doi:10.3389/fonc.2020.00348)
Supplement: Supplementary file 1 [file Data_Sheet_1.DOCX]

**Supplementary Figure**


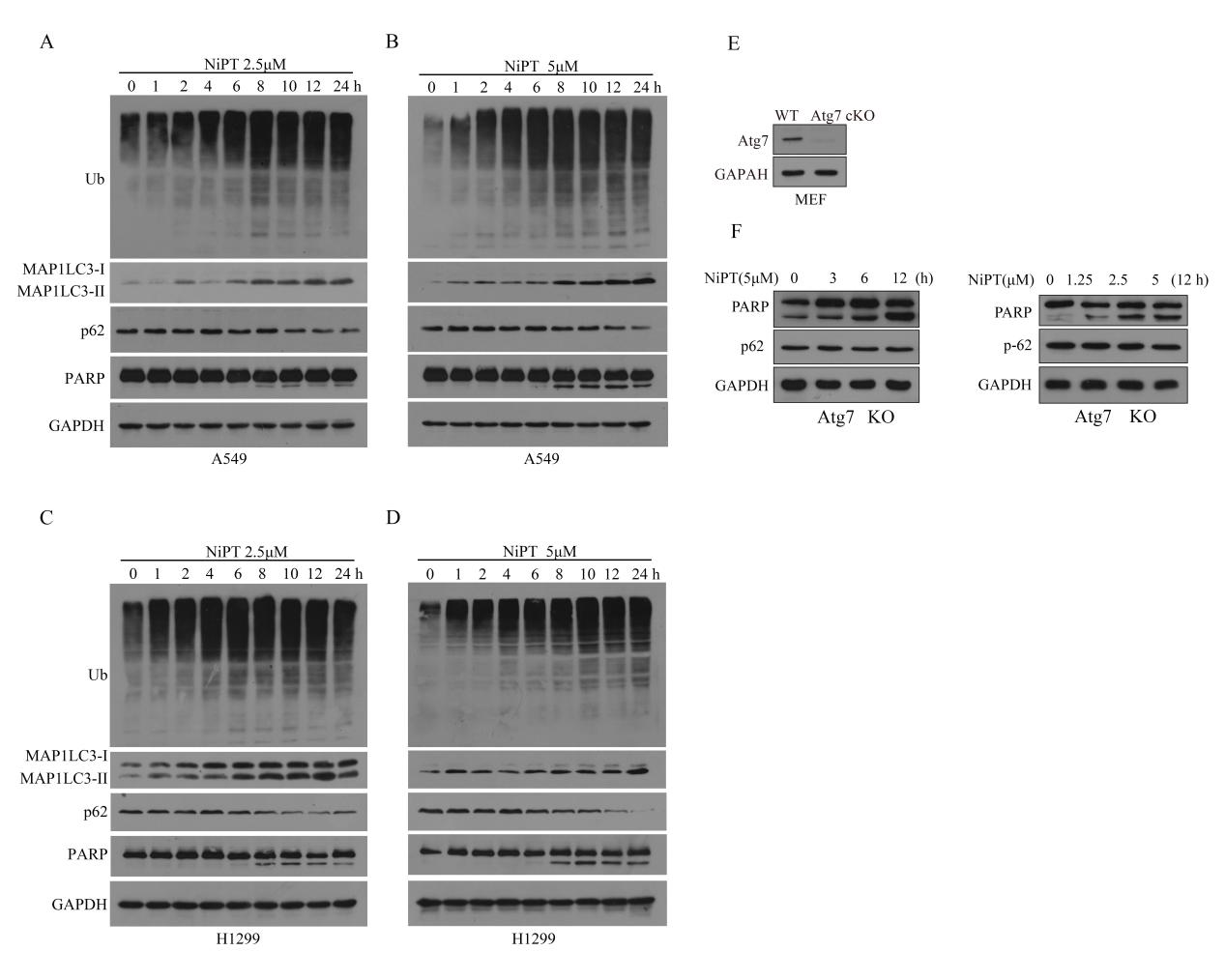
Figure 1.

(A) A549 cells were treated with 2.5μM NiPT for the various times (1, 2, 4, 6, 8, 10, 12, 24h). The expression levels of Ub, LC3 and p62 protein were analyzed by immunoblotting.

(B) A549 cells were treated with 5μM NiPT for the various times (1, 2, 4, 6, 8, 10, 12, 24h). The expression levels of Ub, LC3 and p62 protein were analyzed by immunoblotting.

(C) H1299 cells were treated with 2.5μM NiPT for the various times (1, 2, 4, 6, 8, 10, 12, 24h). The expression levels of Ub, LC3 and p62 protein were analyzed by immunoblotting.

(D) H1299 cells were treated with 5μM NiPT for the various times (1, 2, 4, 6, 8, 10, 12, 24h). The expression levels of Ub, LC3 and p62 protein were analyzed by immunoblotting.

(E) Atg7 KO cells was confirmed by western blot analysis.

(F) Atg7 KO cells were treated with 5 μM NiPT for the various times (3, 6, 12 h) with various concentrations of NiPT for 12 h. The expression levels of PARP, LC3 and p62 protein were analyzed by immunoblotting.
